# Correction: Impact of self-imposed prevention measures and short-term government-imposed social distancing on mitigating and delaying a COVID-19 epidemic: A modelling study

**DOI:** 10.1371/journal.pmed.1003499

**Published:** 2020-12-04

**Authors:** The PLOS Medicine Staff

There were transcription errors which resulted in incorrect rendering of some symbols in Table 1. Please see the corrected Table 1. The publisher apologizes for the error.

**Table 1:** Parameter values for the transmission model with and without awareness.

Parameters		Value*	Source
**Epidemiological parameters**			
Basic reproduction number	*R*_0_	2.5 (2–3)	Li and colleagues [5], Park and colleagues [30], sensitivity analyses
Probability of transmission per contact with *I*_*S*_	*ε*	0.048	From *R*_0_ = *β*[*pσ*/*γ*_*M*_+(1−*p*)/ν]
Transmission rate of infection via contact with *I*_*S*_	*β*	0.66 per day	*β* = *cε*
Average contact rate (unique persons)	*c*	13.85 persons per day	Mossong and colleagues [31]
Relative infectivity of infectious with mild disease (*I*_*M*_)	*σ*	50% (25%–75%)	Assumed, see, e.g., Liu and colleagues [29], sensitivity analyses
Proportion of infectious with mild disease (*I*_*M*_)	*p*	82% (82%–90%)	Wu and colleagues [32], Anderson and colleagues [20], sensitivity analyses
Delay between infection and onset of infectiousness (latent period)	1/*α*	4 days	Shorter than incubation period [5, 30, 33]
Delay from onset of infectiousness to diagnosis for *I*_*S*_	1/*ν*	5 (3–7) days	Li and colleagues [5], sensitivity analyses
Recovery period of infectious with mild disease (*I*_*M*_)	1/*γ*_*M*_	7 (5–9) days	Li Xingwang^†^, sensitivity analyses
Delay from diagnosis to recovery for unaware diagnosed (*I*_*D*_)	1/*γ*_*S*_	14 days	WHO [34]
Relative infectivity of isolated (*I*_*D*_)		0%	Assuming perfect isolation
Case fatality rate of unaware diagnosed (*I*_*D*_)	*f*	1.6%	Althaus and colleagues [35] Park and colleagues [30]
Disease-associated death rate of unaware diagnosed (*I*_*D*_)	*η*	0.0011 per day	*η* = *γ*_*S*_*f*/(1−*f*)
**Awareness parameters**			
Rate of awareness spread (slow, fast and range)	*δ*	5×10^−5^, 1 (10^−6^ − 1) per year	Assumed, sensitivity analyses
Relative susceptibility to awareness acquisition for *S*, *E*, *I*_*M*_, and *R*_*M*_	*k*	50% (0%–100%)	Assumed, sensitivity analyses
Duration of awareness for *S*^*a*^, *E*^*a*^, $I_{M}^{a}$, and $R_{M}^{a}$	1/*μ*	30 (7–365) days	Assumed, sensitivity analyses
Duration of awareness for $I_{S}^{a}$	1/*μ*_*S*_	60 (7–365) days	Longer than 1/*μ*, sensitivity analyses
Delay from onset of infectiousness to diagnosis for $I_{S}^{a}$	1/*ν*^*a*^	3 (1–5) days	Shorter than 1/*ν*, sensitivity analyses
Delay from diagnosis to recovery of aware diagnosed ($I_{D}^{a}$)	$1/\gamma_{S}^{a}$	12 days	Shorter than 1/*γ*_*S*_
Case fatality rate of aware diagnosed ($I_{D}^{a}$)	*f*^*a*^	1%	Smaller than *f*
Disease-associated death rate of aware diagnosed ($I_{D}^{a}$)	*η*^*a*^	0.0008 per day	$\eta =\gamma_{S}^{a}f^{a}/(1-f^{a})$
**Prevention measure parameters**			
Efficacy of mask-wearing (reduction in infectivity)		0%–100%	Varied
Efficacy of handwashing (reduction in susceptibility)		0%–100%	Varied
Efficacy of self-imposed contact rate reduction		0%–100%	Varied
Efficacy of government-imposed contact rate reduction		0%–100%	Varied
Duration of government-imposed social distancing		3 (1–13) months	Assumed, sensitivity analyses
Threshold for initiation of government-imposed social distancing		10 (1–1,000) diagnoses	Assumed, sensitivity analyses

*Mean or median values were used from literature; range was used in the sensitivity analyses.

^†^Expert at China's National Health Commission.
