# Supplementary material for: Impact of self-imposed prevention measures and short-term government-imposed social distancing on mitigating and delaying a COVID-19 epidemic: A modelling study
Source: PLoS Med. 2020 Jul 21;17(7):e1003166. doi: 10.1371/journal.pmed.1003166 (PMC7373263; doi:10.1371/journal.pmed.1003166)
Supplement: S1 Text — (PDF) [file pmed.1003166.s001.pdf]

Mathematical description of the model

1 Baseline transmission model

We developed a deterministic compartmental model describing SARS-CoV-2 transmission in a population stratified by disease status (see Figure 1 in the main text). In the baseline model, individuals are classified as susceptible (S), latently infected (E), infectious with mild disease (I_M), infectious with severe disease (I_S), diagnosed and isolated (I_D), and recovered (R_M and R_S after mild or severe disease, respectively). Susceptible individuals (S) become latently infected (E) through contact with infectious individuals (I_M and I_S) with the force of infection λ_{inf} dependent on the fractions of the population in I_M and I_S compartments. Latently infected individuals (E) become infectious at a rate α; a proportion p of the latently infected individuals will go to the I_M compartment, a proportion $(1-p)$ to the I_S compartment. We assume that infectious individuals with mild disease (I_M) do not require medical attention and recover (R_M) with rate γ_M without being conscious of having contracted COVID-19. Infectious individuals with severe disease (I_S) are unable to recover without medical help, and subsequently get diagnosed and isolated (I_D) with rate ν (in e.g. hospitals, long-term care facilities, nursing homes) and know or suspect they have COVID-19 when they are detected. Therefore, the diagnosed compartment I_D contains infectious individuals with severe disease who are both officially diagnosed and get treatment in healthcare institutions and are not officially diagnosed but have a disease severe enough to suspect they have COVID-19 and require treatment as well as isolation. For simplicity, isolation of these individuals is assumed to be perfect until recovery (R_S) which occurs at rate γ_S, and, hence, they neither contribute to transmission nor to the contact process. Given the timescale of the epidemic and the lack of reliable reports on reinfections, we assume that recovered individuals (R_M and R_S) cannot be reinfected. The infectivity of infectious individuals with mild disease is lower by a factor $0 \leq \sigma \leq 1$ than the infectivity of infectious individuals with severe disease [1]. Natural birth and death processes are neglected as the time scale of the epidemic is short compared to the mean life span of individuals. However, isolated infectious individuals with severe disease (I_D) may be removed from the population due to disease-associated mortality at rate η.

The transmission model without awareness is given by the following system of ordinary differential equations

$$\begin{aligned}
\frac{\mathrm{d}S(t)}{\mathrm{d}t} &= -S(t)\lambda_{\mathrm{inf}}(t)\\
\frac{\mathrm{d}E(t)}{\mathrm{d}t} &= S(t)\lambda_{\mathrm{inf}}(t) - \alpha E(t)\\
\frac{\mathrm{d}I_M(t)}{\mathrm{d}t} &= p\alpha E(t) - \gamma_M I_M(t)\\
\frac{\mathrm{d}I_S(t)}{\mathrm{d}t} &= (1-p)\alpha E(t) - \nu I_S(t)\\
\frac{\mathrm{d}I_D(t)}{\mathrm{d}t} &= \nu I_S(t) - \gamma_S I_D(t) - \eta I_D(t)\\
\frac{\mathrm{d}R_M(t)}{\mathrm{d}t} &= \gamma_M I_M(t)\\
\frac{\mathrm{d}R_S(t)}{\mathrm{d}t} &= \gamma_S I_D(t),
\end{aligned} \tag{1}$$

where

$$\lambda_{\mathrm{inf}}(t) = \frac{\beta}{N(t)}\left[\sigma I_M(t) + I_S(t)\right] \tag{2}$$

is the force of infection and $N(t) = S(t) + E(t) + I_M(t) + I_S(t) + R_M(t) + R_S(t)$ is the total number of individuals who participate in the contact process.

2 Transmission model with disease awareness

In the extended model with awareness, the population is stratified not only by the disease status but also by the awareness status into disease-aware (S^a, E^a, I_M^a, I_S^a, I_D^a, and R_M^a) and disease-unaware (S, E, I_M, I_S, and R_M) (Figure 2 A in the main text). Disease awareness is a state that can be acquires as well as lost. Disease-aware individuals are distinguished from unaware individuals in two essential ways. First, infectious individuals with severe disease who are disease-aware (I_S^a) get diagnosed and isolated faster (I_D^a) with rate ν^a, stay in isolation for a shorter period of time (recovery rate γ_S^a) and have lower disease-associated mortality (rate η^a) than the same category of unaware individuals. The assumption we make here is that disease-aware individuals (I_S^a) recognize they may have COVID-19 on average faster than disease-unaware individuals (I_S) and get medical help earlier which leads to a better prognosis of I_D^a individuals as compared to I_D individuals. Second, disease-aware individuals are assumed to use self-imposed measures such as handwashing, mask-wearing and self-imposed social distancing that can lower their susceptibility, infectivity and/or contact rate. Individuals who know or suspect their disease status (I_D, I_D^a and R_S) do not adapt any such measures since they assume that they cannot contract the disease again. Hence, they are excluded from the awareness transition process and their behaviour in the contact process is identical to disease-unaware individuals.

A schematic representation of the awareness dynamics is given in Figure 2 B in the main text. Individuals of type

S, E, I_M, I_S, and R_M become aware of the disease with the awareness acquisition rate $\lambda_{\text{aware}}(t)$ proportional to the current number of diagnosed individuals via information shared by the government or media

$$\lambda_{\text{aware}}(t) = \delta \cdot \left[I_D(t) + I_D^a(t)\right],$$

where δ is a constant which describes how fast unaware individuals become aware per unit of time. This formulation is based on Eq. (7) in Perra et al. [2].

We assume that awareness fades and individuals return to the unaware state at a constant rate. The latter means that they no longer use self-imposed measures. We propose that awareness acquisition and fading rates are the same for individuals who are susceptible (S), latently infected (E), infectious with mild disease (I_M) and recovered after mild disease (R_M). The rate of awareness acquisition for these individuals is a factor $0 \leq k \leq 1$ lower than the rate of awareness acquisition for infectious individuals with severe disease (I_S). Also, infectious individuals with severe disease are more cautious and, therefore, lose awareness at a slower rate than other individuals. Thus, we use μ to denote the decay rate in compartments S^a, E^a, I_M^a, and R_M^a and μ_S for compartment I_S^a, such that $\mu > \mu_S$.

The difference in disease severity and state of awareness affects the transmission rates and we define the following matrix to summarize transmission rates between different types of susceptible and infectious individuals

$$M(t) = \begin{array}{c} \\ \textit{unaware } S \\ \textit{aware } S \end{array} \begin{array}{cccc} \textit{unaware } I_M & \textit{unaware } I_S & \textit{aware } I_M & \textit{aware } I_S \\ \end{array} \left[\begin{array}{cccc} M_{11}(t) & M_{12}(t) & M_{13}(t) & M_{14}(t) \\ M_{21}(t) & M_{22}(t) & M_{23}(t) & M_{24}(t) \end{array}\right]. \tag{3}$$

Here $[M]_{11}$ captures transmission of infection from unaware I_M to unaware S, $[M]_{12}$ from unaware I_S to unaware S, $[M]_{13}$ from aware I_M to unaware S, $[M]_{14}$ from from aware I_M to unaware S. Similarly, the second row of the matrix captures transmission of infection to susceptible individuals who are aware, S^a. To sum up,

$$S + I_M \xrightarrow{[M]_{11}} E + I_M \ , \ S + I_S \xrightarrow{[M]_{12}} E + I_S$$
$$S + I_M^a \xrightarrow{[M]_{13}} E + I_M^a \ , \ S + I_S^a \xrightarrow{[M]_{14}} E + I_S^a$$
$$S^a + I_M \xrightarrow{[M]_{21}} E^a + I_M \ , \ S^a + I_S \xrightarrow{[M]_{22}} E^a + I_S$$
$$S^a + I_M^a \xrightarrow{[M]_{23}} E^a + I_M^a \ , \ S^a + I_S^a \xrightarrow{[M]_{24}} E^a + I_S^a.$$

The transmission model with awareness is given by the following system of ordinary differential equations

$$
\begin{aligned}
\frac{\mathrm{d}S(t)}{\mathrm{d}t} &= -S(t)\lambda_{\text{inf}}(t) - kS(t)\lambda_{\text{aware}}(t) + \mu S^a(t) \\
\frac{\mathrm{d}E(t)}{\mathrm{d}t} &= S(t)\lambda_{\text{inf}}(t) - \alpha E(t) - kE(t)\lambda_{\text{aware}}(t) + \mu E^a(t) \\
\frac{\mathrm{d}I_M(t)}{\mathrm{d}t} &= p\alpha E(t) - \gamma_M I_M(t) - kI_M(t)\lambda_{\text{aware}}(t) + \mu I_M^a(t) \\
\frac{\mathrm{d}I_S(t)}{\mathrm{d}t} &= (1-p)\alpha E(t) - \nu I_S(t) - I_S(t)\lambda_{\text{aware}}(t) + \mu_S I_S^a(t) \\
\frac{\mathrm{d}I_D(t)}{\mathrm{d}t} &= \nu I_S(t) - \gamma_S I_D(t) - \eta I_D(t) \\
\frac{\mathrm{d}S^a(t)}{\mathrm{d}t} &= -S^a(t)\lambda_{\text{inf}}^a(t) + kS(t)\lambda_{\text{aware}}(t) - \mu S^a(t) \\
\frac{\mathrm{d}E^a(t)}{\mathrm{d}t} &= S^a(t)\lambda_{\text{inf}}^a(t) - \alpha E^a(t) + kE(t)\lambda_{\text{aware}}(t) - \mu E^a(t) \\
\frac{\mathrm{d}I_M^a(t)}{\mathrm{d}t} &= p\alpha E^a(t) - \gamma_M I_M^a(t) + kI_M(t)\lambda_{\text{aware}}(t) - \mu I_M^a(t) \\
\frac{\mathrm{d}I_S^a(t)}{\mathrm{d}t} &= (1-p)\alpha E^a(t) - \nu^a I_S^a + I_S(t)\lambda_{\text{aware}}(t) - \mu_S I_S^a(t) \\
\frac{\mathrm{d}I_D^a(t)}{\mathrm{d}t} &= \nu^a I_S^a(t) - \gamma_S^a I_D^a(t) - \eta^a I_D^a(t) \\
\frac{\mathrm{d}R_M(t)}{\mathrm{d}t} &= \gamma_M I_M(t) - kR_M(t)\lambda_{\text{aware}}(t) + \mu R_M^a(t) \\
\frac{\mathrm{d}R_M^a(t)}{\mathrm{d}t} &= \gamma_M I_M^a(t) + kR_M(t)\lambda_{\text{aware}}(t) - \mu R_M^a(t) \\
\frac{\mathrm{d}R_S(t)}{\mathrm{d}t} &= \gamma_S I_D(t) + \gamma_S^a I_D^a(t),
\end{aligned}
\tag{4}
$$

where

$$\lambda_{\text{aware}}(t) = \delta \cdot [I_D(t) + I_D^a(t)] \tag{5a}$$

$$\lambda_{\text{inf}}(t) = [M(t)]_{11} I_M(t) + [M(t)]_{12} I_S(t) + [M(t)]_{13} I_M^a(t) + [M(t)]_{14} I_S^a(t) \tag{5b}$$

$$\lambda_{\text{inf}}^a(t) = [M(t)]_{21} I_M(t) + [M(t)]_{22} I_S(t) + [M(t)]_{23} I_M^a(t) + [M(t)]_{24} I_S^a(t). \tag{5c}$$

For the population where disease-aware individuals do not use self-imposed measures matrix M takes the following form

$$M_0(t) = \frac{\beta}{N_T(t)} \begin{bmatrix} \sigma & 1 & \sigma & 1 \\ \sigma & 1 & \sigma & 1 \end{bmatrix} \tag{6}$$

with $N_T(t) = S(t) + E(t) + I_M(t) + I_S(t) + S^a(t) + E^a(t) + I_M^a(t) + I_S^a(t) + R_M(t) + R_M^a(t) + R_S(t)$.

Estimates of epidemiological parameters were obtained from previous studies and are shown in Table 1 in the main text.

3 Prevention measures

We considered short-term government intervention aimed at fostering social distancing in the population and a suite of measures self-imposed by disease-aware individuals, i.e., mask-wearing, handwashing, and self-imposed social distancing.

3.1 Mask-wearing

Mask-wearing does not reduce the individual's susceptibility because laypersons, i.e., not medical professionals, are unfamiliar with correct procedures for its use and may often engage in face-touching and mask adjustment. [3] The efficacy of mask wearing is described by a reduction in infectivity of disease-aware infectious individuals (I_S^a and I_M^a) and is represented by a factor r_1, $0 \leq r_1 \leq 1$. The respective transmission matrix is given by

$$M_1 = \frac{\beta}{N_T(t)} \begin{bmatrix} \sigma & 1 & r_1\sigma & r_1 \\ \sigma & 1 & r_1\sigma & r_1 \end{bmatrix} \tag{7}$$

with $N_T(t) = S(t) + E(t) + I_M(t) + I_S(t) + R_M(t) + R_S(t) + S^a(t) + E^a(t) + I_M^a(t) + I_S^a(t) + R_M^a(t)$.

3.2 Handwashing

Since infectious individuals may transmit the virus to others without direct physical contact, we assume that handwashing only reduces one's susceptibility. The efficacy of handwashing is described by a reduction in susceptibility (i.e., probability of transmission of infection per single contact) of susceptible disease-aware individuals (S^a) and is represented by a factor r_2, $0 \leq r_2 \leq 1$. The respective transmission matrix is given by

$$M_2 = \frac{\beta}{N_T(t)} \begin{bmatrix} \sigma & 1 & \sigma & 1 \\ r_2\sigma & r_2 & r_2\sigma & r_2 \end{bmatrix} \tag{8}$$

with $N_T(t) = S(t) + E(t) + I_M(t) + I_S(t) + R_M(t) + R_S(t) + S^a(t) + E^a(t) + I_M^a(t) + I_S^a(t) + R_M^a(t)$.

3.3 Self-imposed social distancing

Disease awareness may also lead individuals to practice social distancing, i.e., maintaining distance to others and avoiding congregate settings. Social distancing of disease-aware individuals is modeled as a reduction in their contact rate. As a consequence, this measure leads to a change in mixing patterns in the population. We model the reduction in contact rate of aware individuals by using the parameter r_3, $0 \leq r_3 \leq 1$. Recall that individuals who recovered from a mild infection may still think of themselves as susceptible, which implies that they are affected by the awareness contagion process. They can, therefore, practice social distancing after they recover. The respective

transmission matrix is given by

$$M_4 = \frac{\beta}{N(t) + r_3 N^a(t)} \begin{bmatrix} \sigma & 1 & r_3\sigma & r_3 \\ r_3\sigma & r_3 & r_3^2\sigma & r_3^2 \end{bmatrix}, \tag{9}$$

where $N(t) = S(t) + E(t) + I_M(t) + I_S(t) + R_M(t) + R_S(t)$ and $N^a(t) = S^a(t) + E^a(t) + I_M^a(t) + I_S^a(t) + R_M^a(t)$.

3.4 Short-term government-imposed social distancing

Governments may decide to promote social distancing policies through interventions such as school and workplace closures, or by issuing a ban on large gatherings and issuing stay-at-home orders [4–7], if the number of diagnosed individuals exceeds a certain threshold. Such a policy will cause a community-wide contact rate reduction, regardless of the awareness status. We model government-imposed social distancing by reducing the average contact rate in the population by a factor r_4, $0 \leq r_4 < 1$. This intervention is initiated if the number of diagnosed individuals is above a certain threshold $\tilde{I}$ (e.g., $10 - 1000$ individuals) and terminates after a fixed period of time, denoted $t_{\text{intervention}}$ (e.g., $1 - 13$ months). As such, we assume that the intervention is implemented early in the epidemic. If t_{start} is the time for which $I_D(t) + I_D^a(t) \geq \tilde{I}$, then the transmission matrix is given by

$$M_5(t) = \frac{\beta}{N_T(t)} \cdot \tilde{r} \cdot \begin{bmatrix} \sigma & 1 & \sigma & 1 \\ \sigma & 1 & \sigma & 1 \end{bmatrix}, \tag{10}$$

where

$$\tilde{r} = \begin{cases} r_4, & \text{if } I_D(t) + I_D^a(t) \geq \tilde{I} \text{ and } t \leq t_{\text{start}} + t_{\text{intervention}} \\ 1, & \text{otherwise} \end{cases}$$

and $N_T(t) = S(t) + E(t) + I_M(t) + I_S(t) + R_M(t) + R_S(t) + S^a(t) + E^a(t) + I_M^a(t) + I_S^a(t) + R_M^a(t)$.

References

[1] Liu Y, Yan LM, Wan L, Xiang TX, Le A, Liu JM, et al. Viral dynamics in mild and severe cases of COVID-19. Lancet Infect Dis. 2020. doi: 10.1016/S1473-3099(20)30232-2.

[2] Perra N, Balcan D, Gonçalves B, Vespignani A. Towards a characterization of behavior-disease models. PLoS ONE. 2011;6(8):1–15.

[3] World Health Organization. Coronavirus disease (COVID-19) advice for the public: When and how to use masks. 2020 [cited 2020 March 13]. Available from: https://www.who.int/emergencies/diseases/novel-coronavirus-2019/advice-for-public/when-and-how-to-use-masks.

[4] Wilder-Smith A, Freedman DO. Isolation, quarantine, social distancing and community containment: pivotal role for old-style public health measures in the novel coronavirus (2019-nCoV) outbreak. J Travel Med. 2020. doi: 10.1093/jtm/taaa020.

[5] Moberly, T. Covid-19: school closures and bans on mass gatherings will need to be considered, says England's CMO. BMJ. 2020;368.

[6] Cauchemez S, Ferguson NM, Wachtel C, Tegnell A, Saour G, Duncan B, et al. Closure of schools during an influenza pandemic. Lancet Infect Dis. 2009;9:473–481.

[7] Jackson C, Vynnycky E, Hawker J, Olowokure B, Mangtani P. School closures and influenza: systematic review of epidemiological studies. BMJ Open. 2013;3(2).
