# Supplementary material for: Impact of self-imposed prevention measures and short-term government-imposed social distancing on mitigating and delaying a COVID-19 epidemic: A modelling study
Source: PLoS Med. 2020 Jul 21;17(7):e1003166. doi: 10.1371/journal.pmed.1003166 (PMC7373263; doi:10.1371/journal.pmed.1003166)
Supplement: S2 Text — (PDF) [file pmed.1003166.s002.pdf]

Impact of awareness process on the probability of infection

We compared changes in the probability of infection for individuals who are aware and who are unaware over the studied period of $T_{max} = 2.5$ years for various scenarios of self-imposed measures and government-imposed social distancing (Figure 1). The probabilities were calculated using the following equations

$$\text{Probability of infection of aware individuals} = 1 - \exp\left[-\int_0^{T_{max}} \lambda_{\text{inf}}(t)\,\mathrm{d}t\right] \tag{1a}$$

$$\text{Probability of infection of unaware individuals} = 1 - \exp\left[-\int_0^{T_{max}} \lambda^a_{\text{inf}}(t)\,\mathrm{d}t\right], \tag{1b}$$

where $\lambda_{\text{inf}}(t)$ and $\lambda^a_{\text{inf}}(t)$ are given by Eq. (5b) and (5c).

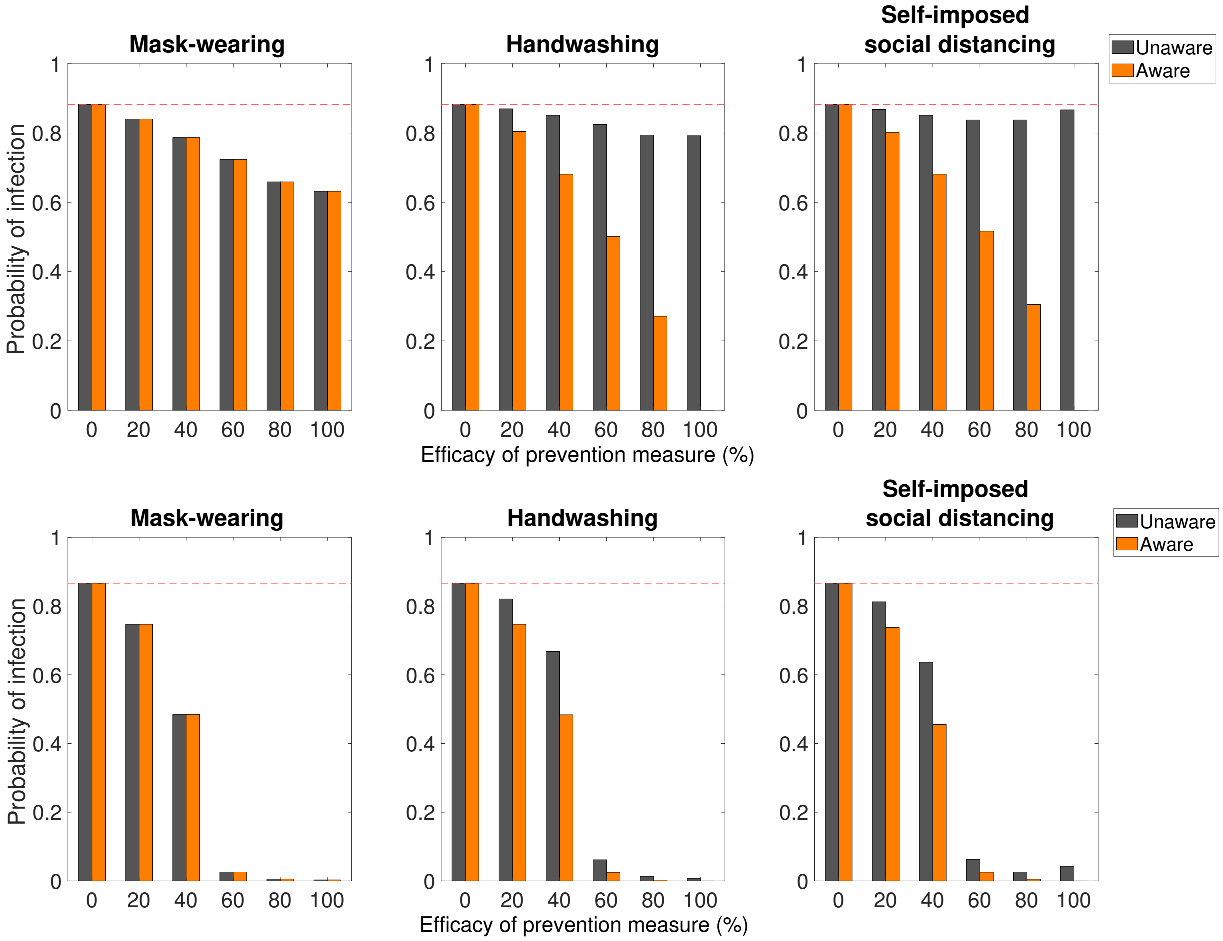

Impact of awareness process on the probability of infection. TOP panels were obtained for a slow rate of awareness spread. **BOTTOM** panels were obtained for a fast rate of awareness spread. The dashed red line indicates probability of infection in the model with awareness and no prevention measures.

We observe that when aware individuals adapt mask wearing, the probability of infection is equally reduced for aware and unaware individuals, as it reduces the infectivity of a part of the population. This measure is most efficient when the rate of awareness spread is fast and infectivity reduction due to mask use is above 40%.

In the case of handwashing, the probability is reduced for both aware and unaware individuals. However, aware

individuals experience a larger reduction. Handwashing yields direct protection to aware individuals, while unaware individuals benefit indirectly from the overall reduced infection level. Similar to mask-wearing, the infection probabilities for both aware and unaware individuals decrease drastically when the efficacy of handwashing exceeds 40% and the rate of awareness spread is fast.

Effects of self-imposed social distancing depend on the rate of awareness spread as well. While aware individuals have reduced probability of infection regardless of the rate of awareness spread, the unaware individuals will only benefit from it when the rate of awareness spread is fast. This is due to modified mixing patterns that emerge as a result of heterogeneous contact rates.

Finally, government-imposed short-term social distancing which lasts for 3 months has no effect on acquisition rates for aware and unaware individuals. The respective probability of infection is marked with dashed red line in Figure 1.
