# Supplementary material for: Impact of self-imposed prevention measures and short-term government-imposed social distancing on mitigating and delaying a COVID-19 epidemic: A modelling study
Source: PLoS Med. 2020 Jul 21;17(7):e1003166. doi: 10.1371/journal.pmed.1003166 (PMC7373263; doi:10.1371/journal.pmed.1003166)
Supplement: S3 Text — (PDF) [file pmed.1003166.s003.pdf]

Sensitivity analyses of the baseline transmission model

To allow for the uncertainty in the parameters of the baseline transmission model, we conducted sensitivity analyses with respect to the proportion of infectious individuals with mild disease (Figure 1), relative infectivity of infectious individuals with mild disease (Figure 2), recovery period of infectious individuals with mild disease (Figure 3), delay from onset of infectiousness to diagnosis for infectious individuals with severe disease (Figure 4), and basic reproduction number (Figure 5). Since our findings in the main text demonstrate that the impact of self-imposed measures is similar across all measures that we considered, we present here only sensitivity analyses for mask-wearing and government-imposed social distancing. All figures were made for a fast rate of awareness spread. In each figure, the panels show the relative reduction in the peak number of diagnoses, the attack rate (proportion of the population that recovered or died after severe infection) and the time until the peak number of diagnoses. In the top row of panels, the efficacy of mask-wearing was varied between 0% and 100%. In the bottom row of panels, the efficacy of government-imposed social distancing was varied between 0% and 100%. In the context of this study, the efficacy of social distancing denotes the reduction in the contact rate. The efficacy of mask-wearing is given by the reduction in infectivity. The simulations were started with one case. Government-imposed social distancing was initiated after 10 diagnoses and lifted after 3 months. For fixed parameter values, see Table 1 in the main text. The parameter which was varied in the sensitivity analyses and the respective range is indicated in each figure.

The main findings of the sensitivity analyses for government-imposed social distancing are

- the time until the peak number of diagnoses does not depend much on any of the explored parameters, except for the basic reproduction number
- the relative reduction in the peak number of diagnoses increases and the attack rate decreases with increasing proportion of infectious individuals with mild disease, decreasing relative infectivity of infectious individuals with mild disease, shorter recovery period of individuals with mild disease and shorter delay from onset of infectiousness to diagnosis for individuals with severe symptoms

The main findings of the sensitivity analyses for mask-wearing are

- for all explored parameter ranges there is a value of efficacy of mask-wearing for which a large epidemic can be prevented
- this critical value of efficacy is very sensitive to the basic reproduction number; smaller value of efficacy is required to prevent a large epidemic for smaller R_0
- the critical value decreases with decreasing relative infectivity of infectious individuals with mild disease and shorter delay from onset of infectiousness to diagnosis for individuals with severe symptoms and is not sensitive to the remaining parameters

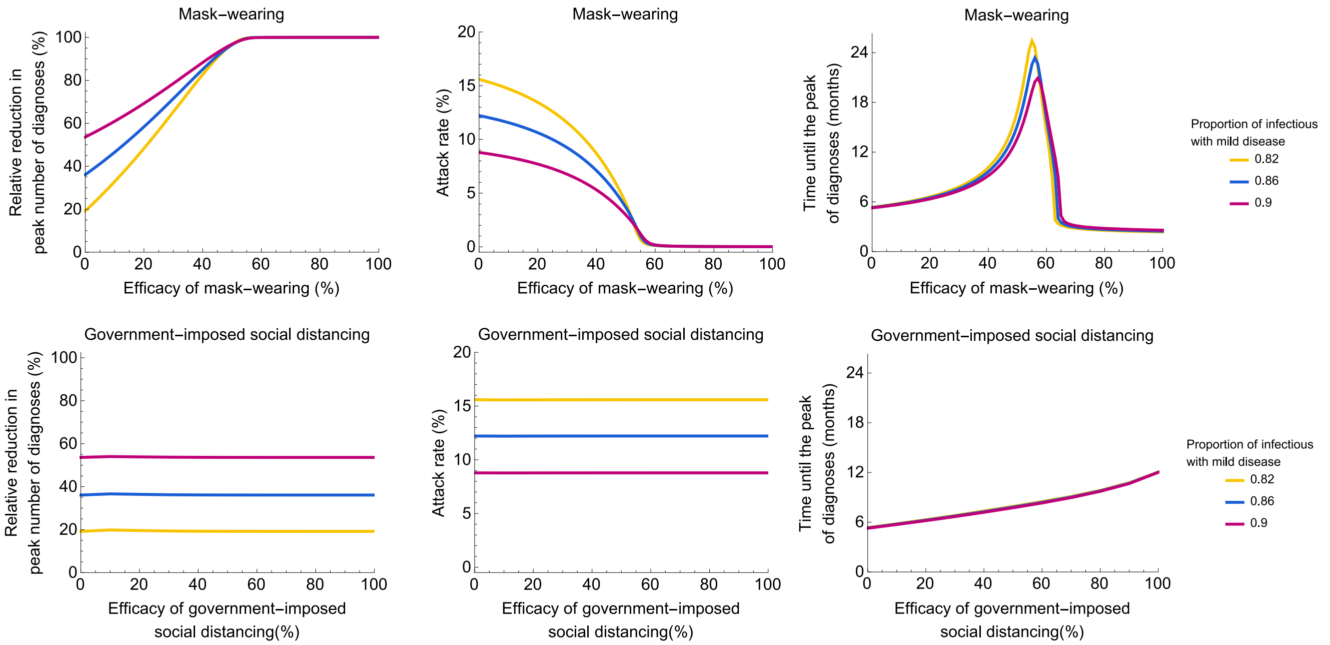

Sensitivity analyses of the baseline transmission model with respect to the proportion of infectious individuals with mild disease.

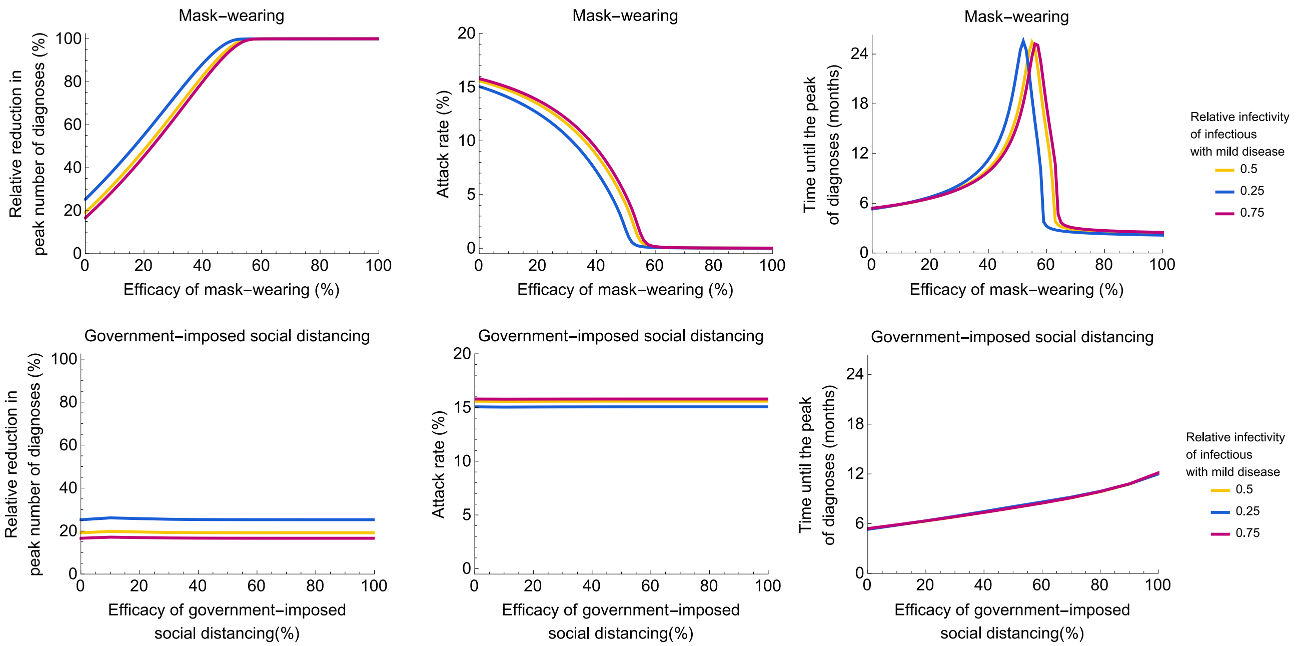

Sensitivity analyses of the baseline transmission model with respect to the relative infectivity of infectious individuals with mild disease.

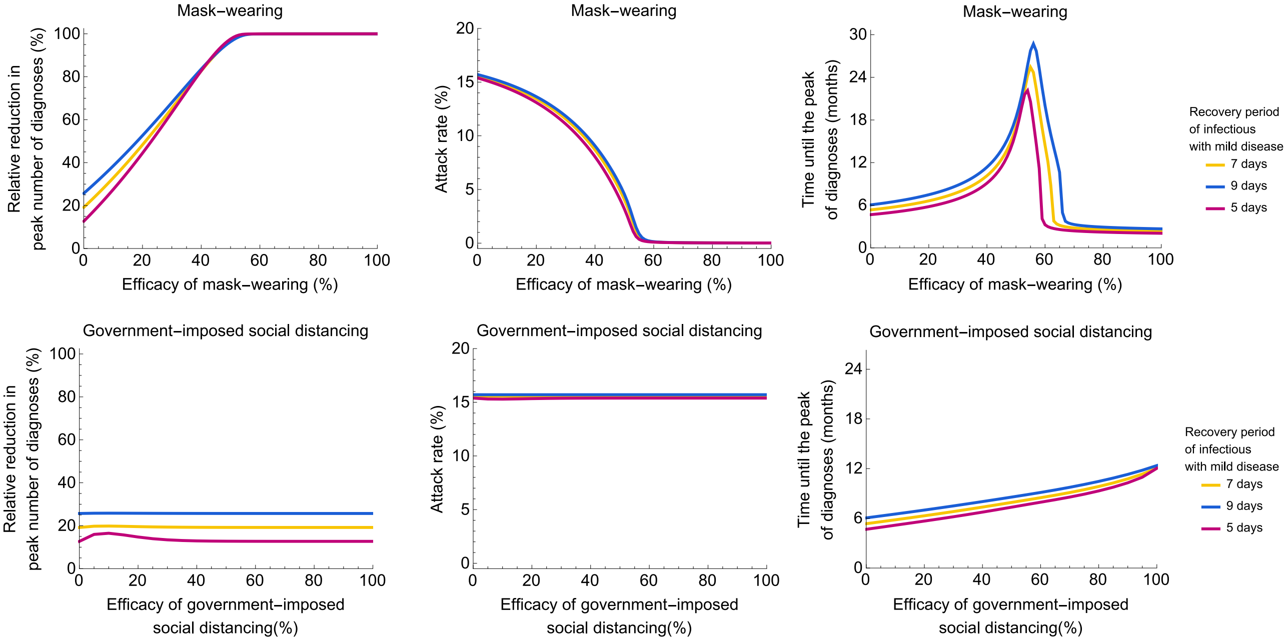

Sensitivity analyses of the baseline transmission model with respect to the recovery period of infectious individuals with mild disease.

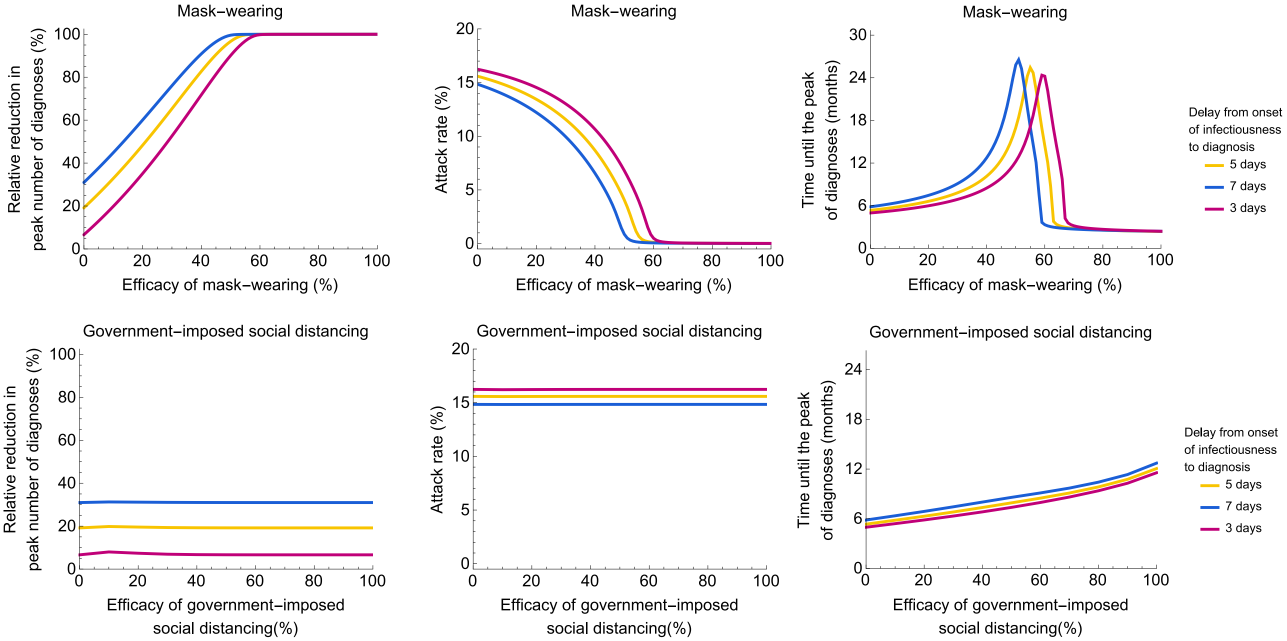

Sensitivity analyses of the baseline transmission model with respect to the delay from onset of infectiousness to diagnosis for infectious individuals with severe disease.

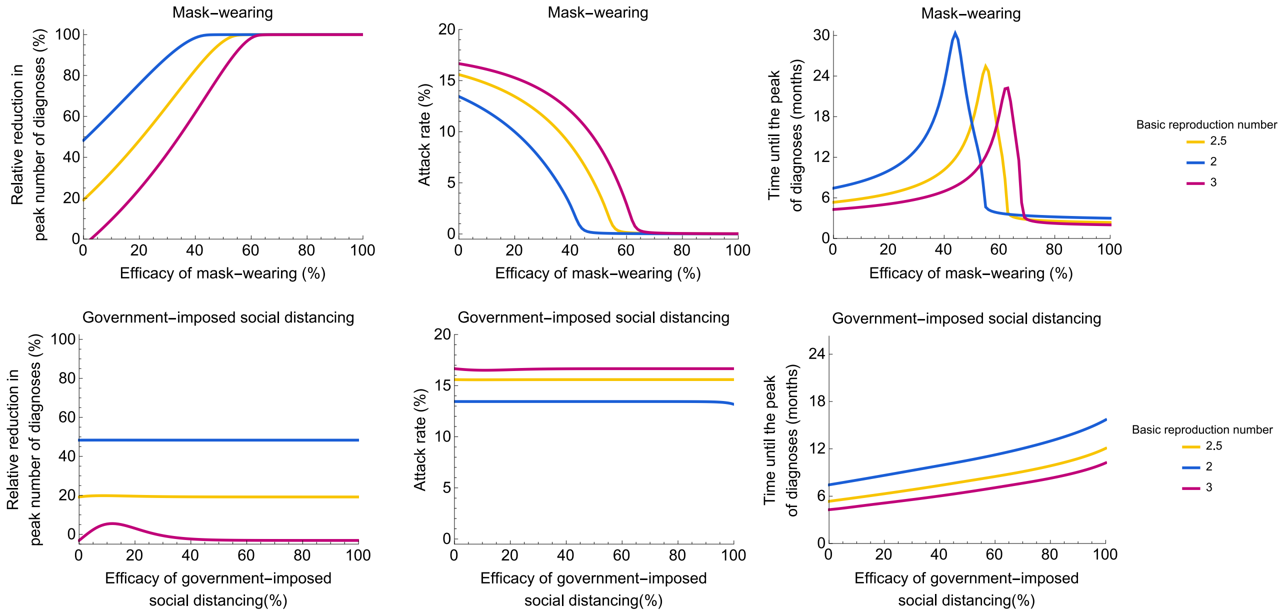

Sensitivity analyses of the baseline transmission model with respect to the basic reproduction number.
