# Supplementary figures and images for: Impact of self-imposed prevention measures and short-term government-imposed social distancing on mitigating and delaying a COVID-19 epidemic: A modelling study
Source: PLoS Med. 2020 Jul 21;17(7):e1003166. doi: 10.1371/journal.pmed.1003166 (PMC7373263; doi:10.1371/journal.pmed.1003166)

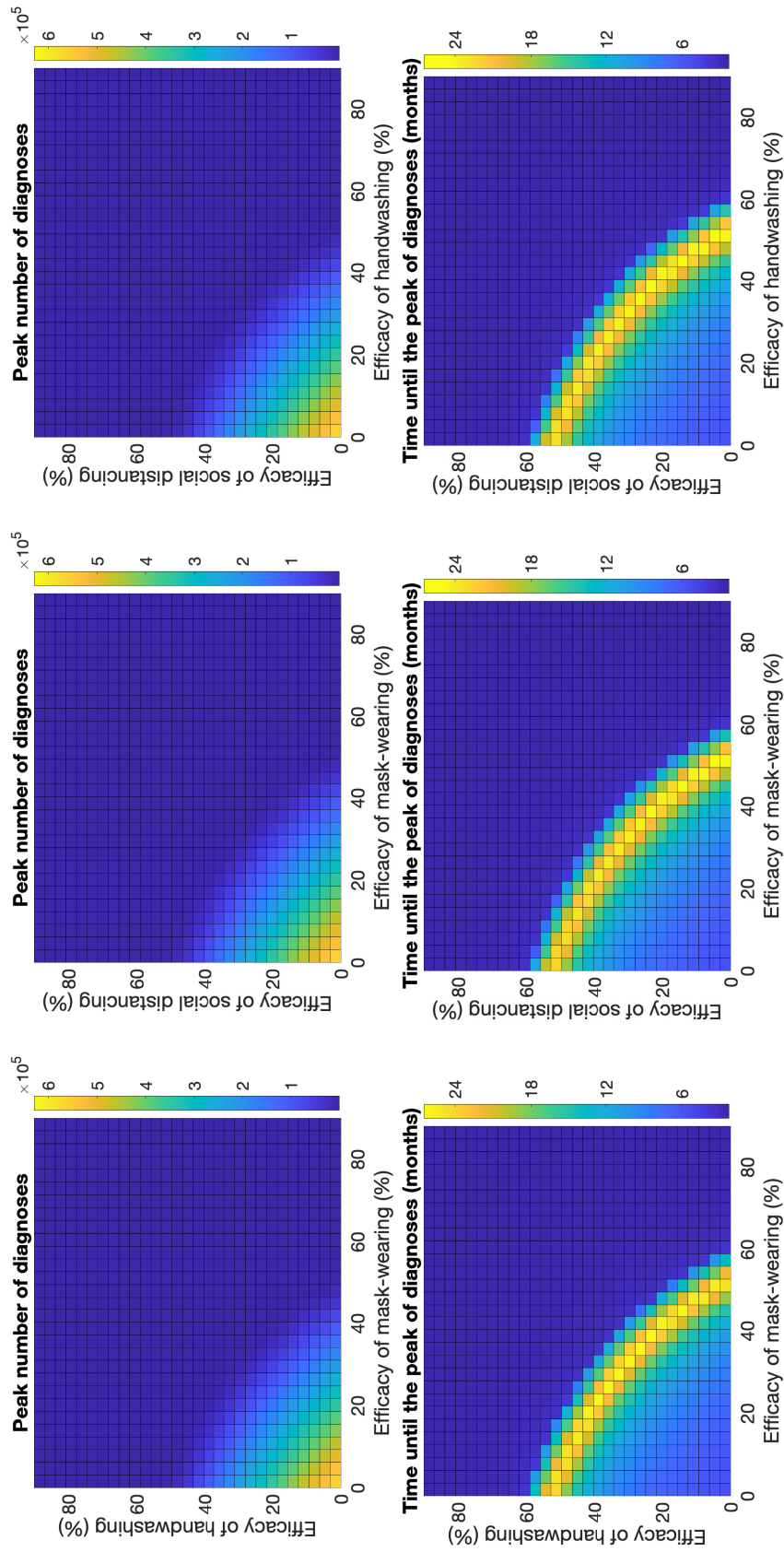

Supplement: S1 Fig — Top and bottom panels show the peak number of diagnoses and time until the peak of diagnoses (months) since the first case for all combinations of self-imposed prevention measures as their efficacy was varied from 0% to 100%. The figures were obtained for a fast rate of awareness spread and population of 17 million individuals. The model predicts that the effect of combinations of self-imposed measures is additive. This means that a large outbreak can be prevented by, for example, a combination of handwashing and self-imposed social distancing, each with an efficacy of around 25% (or other efficacies adding up to 50%). (PDF) [file pmed.1003166.s004.pdf]

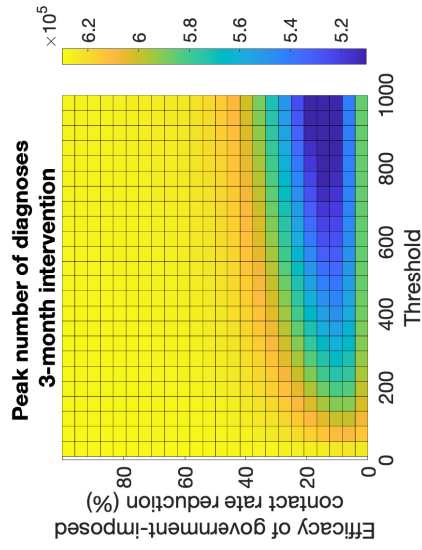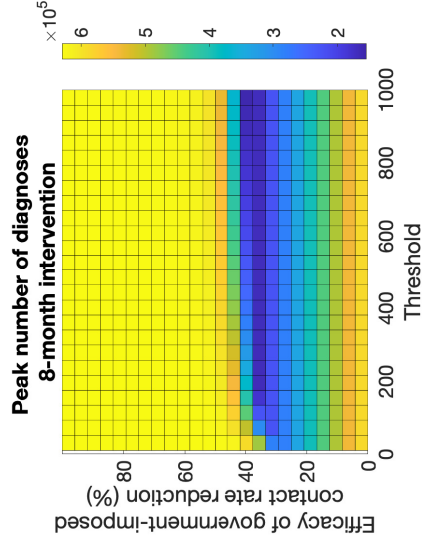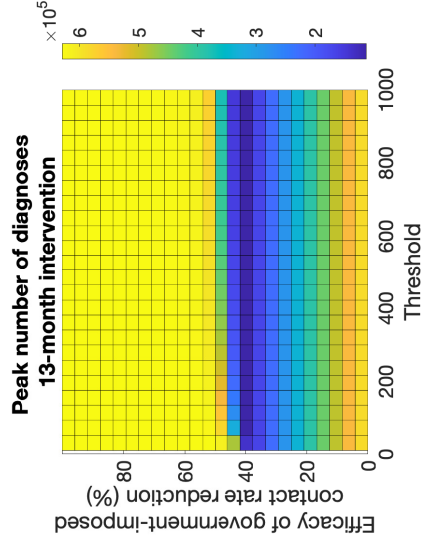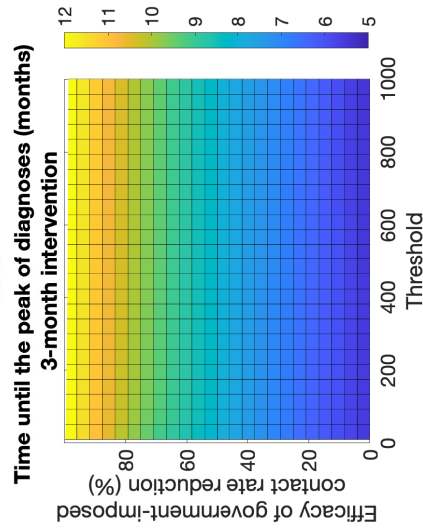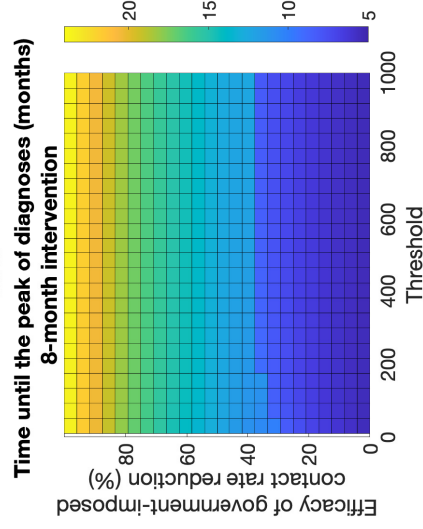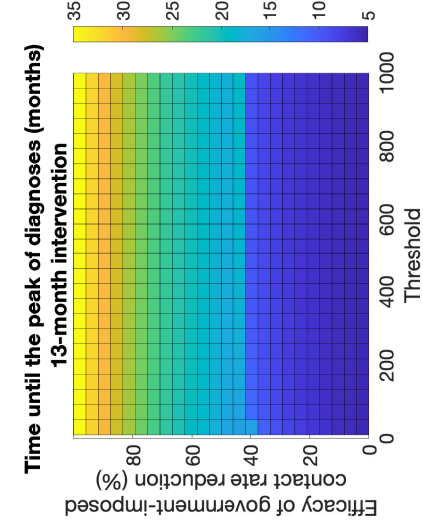

Supplement: S2 Fig — Top and bottom figures show the peak number of diagnoses and time until the peak of diagnoses (months) since the first case for interventions that last 3, 8, and 13 months (left, middle, and right row, respectively). The efficacy of government-imposed contact rate reduction was varied from 0% to 100% (y-axis), and the threshold for initiation of intervention was between 1 and 1,000 diagnoses (x-axis). The figures were obtained for a fast rate of awareness spread and population of 17 million individuals. The model predicts that government intervention introduced later into the epidemic and imposed for a longer period of time not only delays the peak of the epidemic but also reduces it for intermediate efficacy values. (PDF) [file pmed.1003166.s005.pdf]

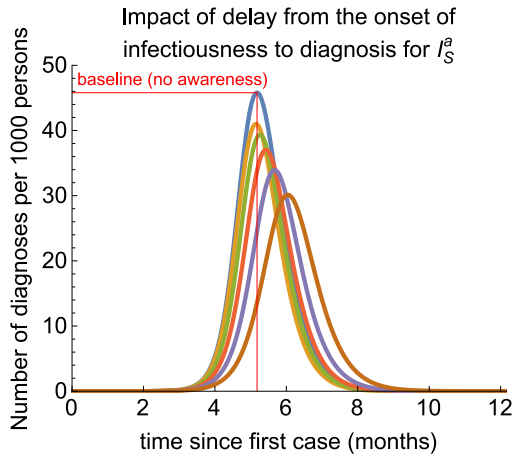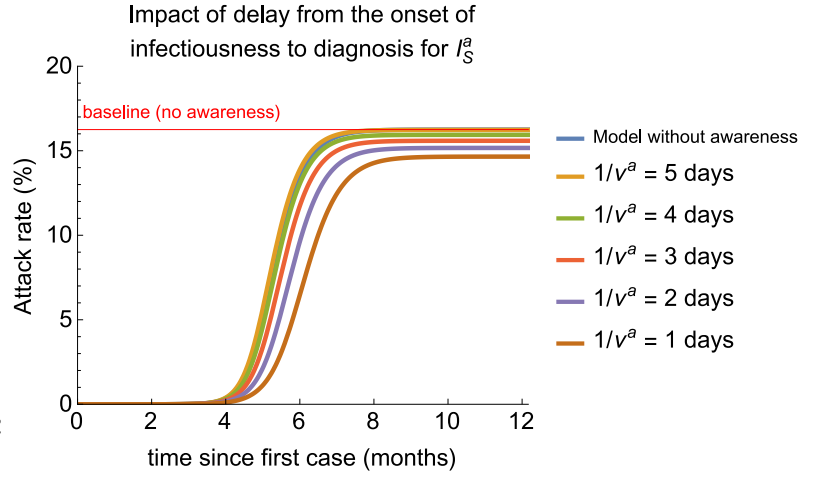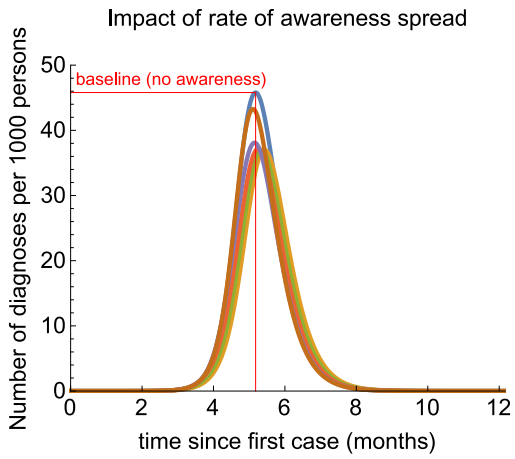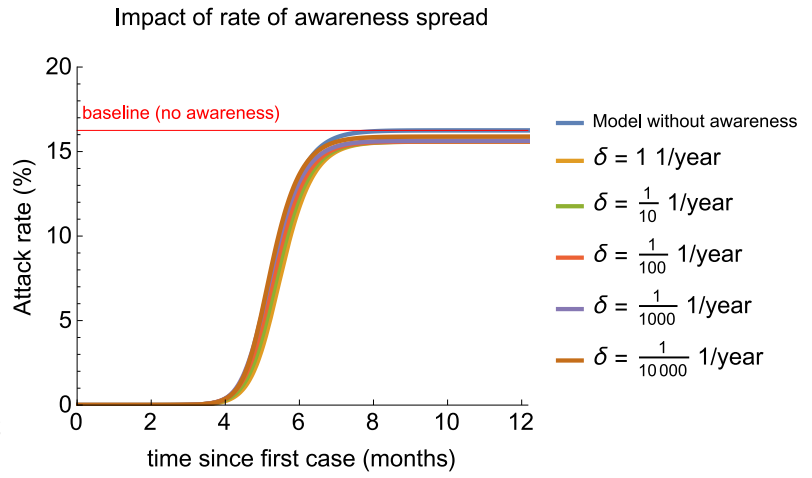

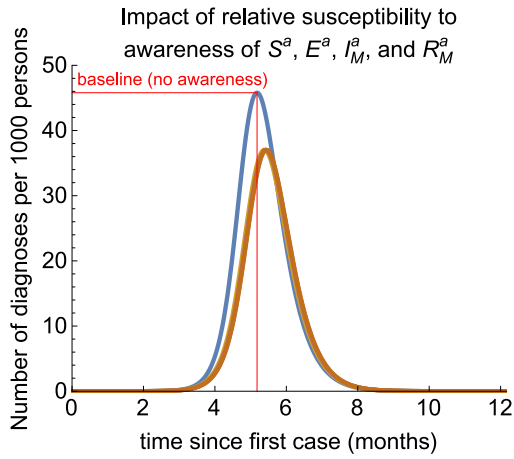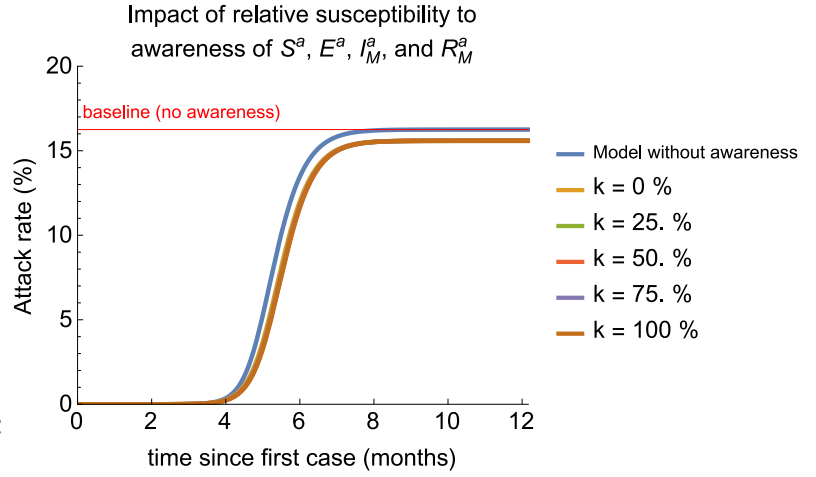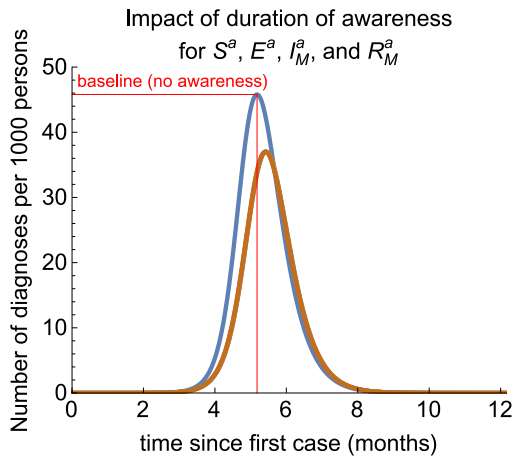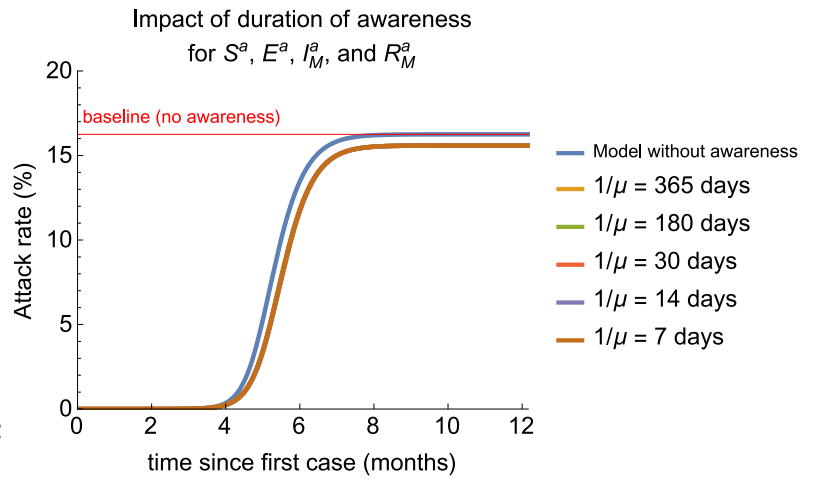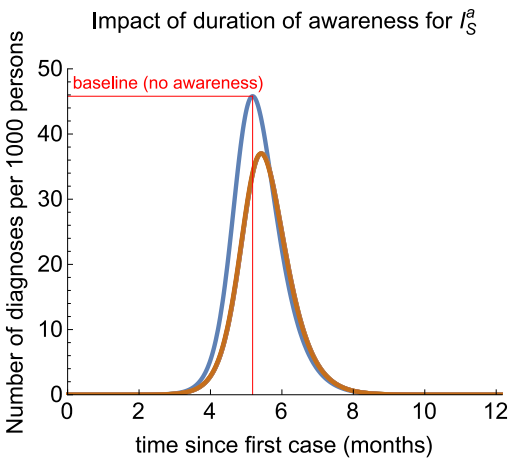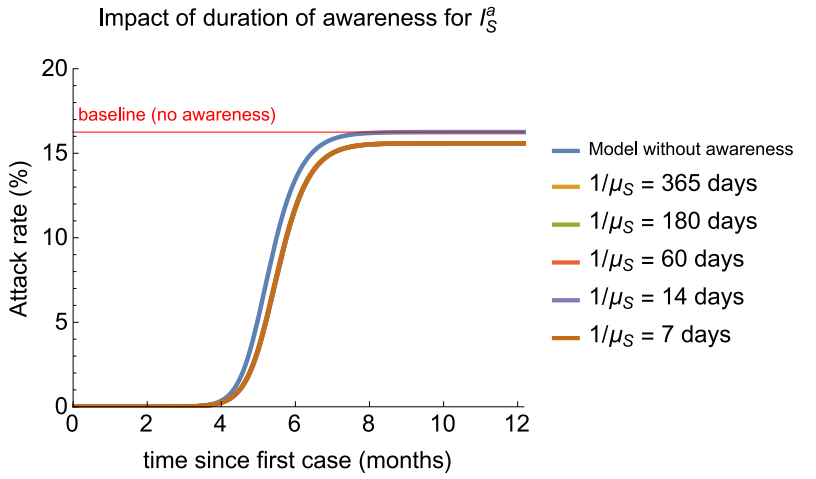

Supplement: S3 Fig — Page 1: The analyses demonstrate that the transmission model with disease awareness is sensitive with respect to changes in the delay from the onset of infectiousness to diagnosis for disease-aware individuals and to the rate of awareness spread. Page 2: The analyses demonstrate that the transmission model with disease awareness is not sensitive with respect to changes in the relative susceptibility to awareness and duration of awareness. (PDF) [file pmed.1003166.s006.pdf]
