# Supplementary material for: Impact of self-imposed prevention measures and short-term government-imposed social distancing on mitigating and delaying a COVID-19 epidemic: A modelling study
Source: PLoS Med. 2020 Jul 21;17(7):e1003166. doi: 10.1371/journal.pmed.1003166 (PMC7373263; doi:10.1371/journal.pmed.1003166)
Supplement: Attachment — Submitted filename: Rebuttal_plosmedicine_reviewer2.pdf [file pmed.1003166.s008.pdf]

Comments from the reviewers

Reviewer #2

Teslya et al write a really interesting piece on the impact of self-imposed and government prevention measures on controlling COVID-19 epidemic. I found the manuscript very well written and the mathematical models carefully described.

We thank the Reviewer for finding our study to be interesting and carefully developed. We addressed your comments one by one below.

I only have minor comments:

1. Abstract:
   Lines 15-17 "Government-imposed social distancing introduced later into the epidemic and kept for a longer period of time not only delays the peak number of diagnoses but also reduces it for intermediate efficacy values" This is not one of the main findings of the article (and it has already been shown in previous articles). This sentence is mentioned only in the Abstract and shown in the Supplementary material but in the main analysis of the paper both timing and duration of government-imposed social distancing are fixed. Therefore, I would suggest removing this sentence from the Abstract.

Response:
We agree with the Reviewer that the quoted sentence is not the main finding of our study. The results for government-imposed social distancing with different thresholds for initiating the intervention (1 to 1000 diagnoses) and different durations (3, 8 and13 months) were added for completeness. This is now mentioned as part of the newly added subsection Sensitivity analyses of the Methods (page 10, lines 213-224), and the sentence has been removed from the Abstract.

2. Author summary:
   Line 26 All figures need to be updated

Response:
On request of the Editor, the Author summary has been formatted according to the PLOS Medicine guidelines. The specific sentence the reviewer mentioned has been rewritten in a way so that it would not require updating the figures but it would align with the current situation and give the motivation for our study (page 3, lines 36-37)

As of May 2020, the coronavirus disease (COVID-19) caused by the novel coronavirus (SARS-CoV-2) has spread to nearly every country in the world since it first emerged in China in December 2019.

Introduction:

3. First paragraph (lines 37-43) needs to be updated using current data

Response:

We updated the first sentence of the Introduction (page 4, lines 62-64) which now reads as follows:

As of May 5, 2020, the novel coronavirus (SARS-CoV-2) has spread worldwide and only 13 countries have not reported any cases. It has caused over 3,640,835 confirmed cases of COVID-19 and nearly 255,100 deaths since the detection of its outbreak in China on December 31, 2019.

4. Line 42 the authors refer to the evidence of pre-symptomatic transmission, but they do not include it in their model. Why? What impact would it have?

Response:
This is a really good question by the Reviewer and we realize that our description of the model could have been better. In fact, the model does include the possibility of fully asymptomatic and pre-symptomatic transmission as exposed (E) individuals enter infectious compartments (I_M and I_S) at the onset of infectiousness rather than at the onset of symptoms. This was described in Table 1 in the original version of the manuscript where we defined the average time spent in the E compartment as the latent period, i.e. time between infection and the onset of infectiousness. The estimate for the latent period was taken to be shorter than currently known incubation period, i.e. time between infection and the onset of symptoms. To make this clear, in the revised manuscript we have added the textual description of latent period in Table 1, changed the description of I_M and I_S compartments in the model diagram (Figure 1) from

Mild symptoms (undiagnosed) → Infectious (mild disease)

Severe symptoms (undiagnosed) → Infectious (severe disease)

We have also made similar changes throughout the text (e.g. as marked on page 5, line 117) and now use the word ‘symptoms’ only where it is appropriate.

5. Lines 46-48 Interventions have included a complete lockdown in several countries, so this sentence needs to be updated/changed

Response:
Since many updates and changes were necessary in the second paragraph of the Introduction, the whole paragraph is now marked in the revised version of the text (page 4, lines 70-82). The modified text reads as follows:

Now that SARS-CoV-2 has extended its range of transmission in all parts of the world, it is evident that many countries face a large COVID-19 epidemic (ECDC2020d). Initial policies regarding COVID-19 prevention were mainly limited to reporting cases, strict isolation of severe symptomatic cases, home isolation of mild cases, and contact tracing (ECDC2020c). However, due to the potentially high contribution of asymptomatic and pre-symptomatic spread (Ferretti 2020), these case-based interventions are likely insufficient in containing a COVID-19 epidemic unless they are highly effective (Hellewell 2020, Ferretti 2020, Fraser 2004, Klinkenberg 2006). Given the rapid rise in cases and the risk of exceeding critical care bed capacities, many countries have implemented social distancing as a short-term measure aiming at reducing the contact rate in the population and, subsequently, transmission (ECDC2020d). Several governments have imposed nationwide partial or complete lockdowns

by closing schools, public places and non-essential businesses, canceling mass events and issuing stay-at-home orders (ECDC2020d).

6. Lines 64-67 please add a simple explanation (few words) of how this could lead to a second wave

Response:
Following your suggestion, we have added the following sentence (pages 4-5, lines 93-95):

The secondary epidemic waves may appear as the result of individuals relaxing adherence to self-imposed measures prematurely in a population where the susceptible pool following the first wave is still significantly large and disease has not been completely eliminated.

Methods

7. Line 97 Has this been shown? Reference is needed here.

Response:
There is some preliminary evidence on this topic. For example, "Liu et al. Viral dynamics in mild and severe cases of COVID-19. Lancet Infectious Diseases. 2020" (https://doi.org/10.1016/S1473-3099(20)30232-2), have shown that the mean viral load of severe cases was around 60 times higher than that of mild cases, suggesting that higher viral loads might be associated with severe clinical outcomes. We have cited this reference as follows (page 6, lines 131-132):

The infectivity of infectious individuals with mild disease is lower than the infectivity of infectious individuals with severe disease (Liu 2020).

In the revised manuscript, we have also included additional sensitivity analyses to account for the uncertainty in this parameter (revised Appendix page 14, Figure 7).

Results

8. Line 189 The probability of infection is mentioned here for the first time. It is currently explained in Table 1 and in the Supplement, so it is worth referring to it.

Response:
Thank you for indicating a gap in our explanation. We have added the probability of infection in the sentence where we describe our model output as follows (page 9, lines 194-196)

The model outputs are the peak number of diagnoses, attack rate (a proportion of the population that recovered or died after severe infection), the time to the peak number of diagnoses since the first case, and the probability of infection during the course of an epidemic (see Appendix for a more detailed description of the latter).

9. Figure 3. I would remove this figure and add a similar one at the end including all scenarios investigated (for one or two choices of efficacies)

Response:
We use Figure 3 as an introduction to explain the analyses we perform. It serves both to showcase the possible effect of awareness and to demonstrate what type of outputs we

consider (peak timing, peak height, attack rate). For this reason, we would like to keep Figure 3. We also believe it is useful to explore the whole range of efficacies in our comparative analyses. Given a specific measure (e.g. handwashing with soap or alcohol-based solution) or a specific setting (e.g. different countries) where adherence to self-imposed measures or stringency of government-imposed social distancing is different, the model would predict different outcomes. On the other hand, in the light of expected easing of lockdowns in many countries, we thought it would be useful to have an additional figure for the combination of government-imposed social distancing and a self-imposed measure (e.g. handwashing) with few choices of efficacies and slow and fast awareness spread. This addition is in line with the suggestion of the Reviewer (see newly added Figure 6 on page 16).

10. Figure 4 panel C the blue line is not visible. Explain in the caption.

Response:
In the revised version of the manuscript we have added the following sentence to the caption of Figure 4 panel C (page 13):

Please note that the blue line corresponding to handwashing is not visible in (C) since it almost completely overlaps with lines for mask-wearing and self-imposed social distancing.

11. Line 208 As this is not shown by the authors, it would be worth adding a ref here

Response:
Thank you for pointing out that this sentence sounded unsupported. The analyses mentioned on line 208 in the original manuscript (i.e. government-imposed social distancing that starts early --- after tens to hundreds of cases --- and is lifted a few weeks to few months later) were performed and included in the original version of the Appendix. To make this clear in the revised manuscript, we have created a new subsection in the Methods where we gave details of the performed sensitivity analyses of the model. The relevant part of it now (page 10, lines 213-224) reads as follows:

In addition, we present results for the impact on the model outcomes […] of the government-imposed social distancing with efficacy ranging from 0% to 100%, different thresholds for initiating the intervention (1 to 1000 diagnoses) and different durations of the intervention (3, 8 and 13 months) (see Appendix for details).

12. Figure 5 Add caption so that the figure can stand alone

Response:
The caption now reads as follows (page 14):

(A), (B) and (C) show the relative reduction in the peak number of diagnoses, the attack rate (proportion of the population that recovered or died after severe infection) and the time until the peak number of diagnoses. The efficacy of prevention measures was varied between 0% and 100%. In the context of this study, the efficacy of social distancing denotes the reduction in the contact rate. The efficacy of handwashing and mask-wearing are given by the reduction in susceptibility and infectivity, respectively. The simulations were started with one case. Government-imposed social distancing was initiated after 10 diagnoses and lifted after 3 months. For parameter values, see Table 1. Please note that the blue line corresponding to

handwashing is not visible in (A) since it almost completely overlaps with lines for mask-wearing and self-imposed social distancing.

Discussion

13. Expected impact of pre-symptomatic transmission

Response:

As we explained in our response to the Reviewer's comment 4), pre-symptomatic transmission is already included in the model. We, therefore, think a further discussion is not necessary.
