# Supplementary material for: Impact of self-imposed prevention measures and short-term government-imposed social distancing on mitigating and delaying a COVID-19 epidemic: A modelling study
Source: PLoS Med. 2020 Jul 21;17(7):e1003166. doi: 10.1371/journal.pmed.1003166 (PMC7373263; doi:10.1371/journal.pmed.1003166)
Supplement: Attachment — Submitted filename: COVID19_PLOSMed.docx [file pmed.1003166.s007.docx]

Teslya et al write a really interesting piece on the impact of self-imposed and government prevention measures on controlling COVID-19 epidemic. I found the manuscript very well written and the mathematical models carefully described.

I only have minor comments:

**Abstract:**

Lines 15-17 *“Government-imposed social distancing introduced later into the epidemic and kept for a longer period of time not only delays the peak number of diagnoses but also reduces it for intermediate efficacy values”* This is not one of the main findings of the article (and it has already been shown in previous articles). This sentence is mentioned only in the Abstract and shown in the Supplementary material but in the main analysis of the paper both timing and duration of government-imposed social distancing are fixed. Therefore, I would suggest removing this sentence from the Abstract.

**Author summary:**

Line 26 All figures need to be updated

**Introduction:**

First paragraph (lines 37-43) needs to be updated using current data

Line 42 the authors refer to the evidence of pre-symptomatic transmission, but they do not include it in their model. Why? What impact would it have?

Lines 46-48 Interventions have included a complete lockdown in several countries, so this sentence needs to be updated/changed

Lines 64-67 please add a simple explanation (few words) of how this could lead to a second wave

**Methods**

Line 97 Has this been shown? Reference is needed here.

**Results**

Line 189 The probability of infection is mentioned here for the first time. It is currently explained in Table 1 and in the Supplement, so it is worth referring to it.

Figure 3. I would remove this figure and add a similar one at the end including all scenarios investigated (for one or two choices of efficacies)

Figure 4 panel C the blue line is not visible. Explain in the caption.

Line 208 As this is not shown by the authors, it would be worth adding a ref here

Figure 5 Add caption so that the figure can stand alone

**Discussion**

Expected impact of pre-symptomatic transmission
